# Semantic Information Theory and Applications

**DOI:** 10.3390/e27111092

**Published:** 2025-10-23

**Authors:** Meixia Tao, Kai Niu, Youlong Wu

**Affiliations:** 1 Department of Electronic Engineering, Shanghai Jiao Tong University, Shanghai 200240, China; mxtao@sjtu.edu.cn; 2 The Key Laboratory of Universal Wireless Communications, Beijing University of Posts and Telecommunications, Beijing 100876, China; niukai@bupt.edu.cn; 3 School of Information Science and Technology, ShanghaiTech University, Shanghai 200240, China

Traditional information theory provides a rigorous foundation for information compression and reliable symbol transmission. However, in emerging applications such as autonomous driving, remote healthcare, and industrial Internet of Things (IoT), the key communication challenge has been shifted from accurate delivery of raw data to the efficient transmission of task-relevant information [1,2,3,4]. For instance, autonomous vehicles require timely awareness of driving-critical events rather than full sensor streams, while remote healthcare relies on essential medical features rather than complete high-resolution images. These task-driven communication scenarios are inherently bandwidth-constrained and latency-sensitive, calling for integrating downstream task objectives into information processing and transmission to improve the communication efficiency.

Semantic communication has emerged as a new paradigm that operates at the semantic or effectiveness level, aiming to extract and convey only the information necessary for a receiver to accomplish a specific goal [5,6,7,8,9,10]. The rapid advancement of artificial intelligence (AI) has significantly accelerated research in this field. By leveraging AI techniques, transmitters can efficiently extract, compress, and encode task-relevant information, thereby reducing communication overhead and enabling more intelligent, goal-oriented transmission. Consequently, semantic communication has attracted increasing attention as a key enabler of next-generation intelligent networks.

Despite the notable progress made in semantic communications, a unified theoretical foundation and operational analytical tools remain elusive. The relationship between traditional information theory and semantic communication is not yet fully established, particularly regarding the extent to which information-theoretic principles can address the unique challenges posed by semantic communication. This Special Issue aims to bridge this gap by promoting the convergence of information theory and semantic communication, thereby fostering deeper theoretical insights and practical advancements. After going through a rigorous review process, twelve articles are selected in this Special Issue, including two review articles and ten research articles. The review articles provide holistic perspectives on the semantic information theory and semantic communications, respectively. The novel contributions in the research articles can be broadly categorized into two directions: semantic rate–distortion theory, which investigates source compression under task-specific semantic distortion measures, and efficient semantic communication system design, which focuses on optimizing transmission efficiency while preserving semantic relevance.

The review article [11] takes a complementary perspective, advocating for a generalization of Shannon’s framework rather than a parallel theory. It introduces a “G theory” that replaces the traditional distortion constraint with a semantic constraint, employing truth functions to quantify semantic distortion, information, and loss. The paper illustrates the broad applicability of this theory across domains ranging from machine learning to statistical physics and further proposes refining Friston’s minimum free energy principle into the maximum information efficiency principle. The review article [12] presents a systematic review of the state-of-the-art semantic communications in the Internet of Vehicles (IoV). It covers key technologies, including semantic information extraction, system architectures, and resource allocation, and demonstrates their effectiveness in traffic perception, intelligent driving, and traffic management. The survey emphasizes the vast potential applications of semantic communications within IoV.

The six papers dedicated to semantic rate distortion theory [13,14,15,16,17,18] collectively push the boundaries of theoretical analysis by introducing new analytical frameworks and algorithms. Specifically, paper [13] proposes a new semantic arithmetic coding (SAC) method for semantic lossless compression. By leveraging the concept of synonymity and constructing synonymous mappings, the SAC method achieves higher compression efficiency for meaning-contained source sequences, approximating the semantic entropy limits. Paper [14] proposes a framework to quantify the rate-distortion tradeoff for semantic communication under various task-specific semantic distance measures. The authors derive closed-form expressions for the semantic rate-distortion functions for classification and signal generation tasks, providing theoretical results verified by extensive experiments. Paper [15] integrates the perceptual dimension into the rate-distortion paradigm. This work clarifies the role of perceptual constraints common and private randomness and projects an increase in expected traffic in intelligent communication networks when perceptual quality is considered. The paper demonstrates how a modest increase in rate can significantly enhance the perceptual quality of reconstructions. Paper [16] extends the fundamental BA algorithm to the semantic domain. It introduces the Extended Blahut–Arimoto (EBA) algorithm, which iteratively computes the semantic rate-distortion function based on synonymous mapping. The paper also proposes an optimization framework combining EBA with simulated annealing to handle scenarios with unknown synonymous mappings and analyzes the role of the semantic knowledge base size. Paper [17] characterizes the optimal transition probability distribution for reproducing an intrinsic state in a composite source model. The paper interprets this optimal solution as a “soft classifier” that generalizes the traditional “classify-then-compress” scheme. It demonstrates that this approach, which uses soft classification to guide lossy compression, outperforms existing coding schemes. Paper [18] addresses a distributed source coding problem relevant to task-oriented semantic communication. It characterizes the exact rate-distortion function for scenarios where a central decoder aims to recover a latent variable from independently encoded sources. The paper also develops a distributed Blahut–Arimoto (BA) algorithm to numerically compute the rate-distortion function.

The four papers on theoretically guided semantic communication scheme designs [19,20,21,22] demonstrate the practical application of these ideas. Specifically, paper [19] proposes a new semantic communication system for image transmission that addresses bandwidth limitations. The system uses a non-uniform quantization technique based on the differential impact of features on image recovery at the receiver. This dynamic bit allocation algorithm ensures image reconstruction quality under limited bandwidth. Paper [20] fills a key gap by providing an operational interpretation of partial information decomposition. It connects this decomposition to the capacity of the broadcast channel, rigorously interpreting the synergistic information as a cooperative gain. This provides a clear theoretical basis for the technique’s applications. Paper [21] proposes a separate source–channel coding framework for semantic communications in MIMO systems, improving adaptability and model reusability compared to joint source–channel coding. The framework combines a VAE-based semantic coder, a communication-informed bottleneck attribution for feature importance, and an importance-aware resource allocation scheme. Ref. [22] proposes a task-oriented framework for multimodal digital semantic communications. It leverages a pre-trained transformer to extract semantic information and designs three semantic source-coding schemes: quantization-based coding, compression via clustering, and a vector-quantized autoencoder-based learned codebook. By combining these semantic codes with standard channel coding, the study demonstrates that the proposed approaches achieve comparable classification accuracy while significantly improving system time efficiency over learning-based baselines.

This Special Issue underscores the pivotal role of information theory in shaping the emerging paradigm of semantic communication. The collected articles demonstrate how classical tools—most notably rate–distortion theory and joint source–channel coding—can be extended to support more efficient and reliable approaches to data compression and information transmission. Information theory thus provides indispensable theoretical guidance for the advancement of semantic communication, while the rapid progress of semantic communication simultaneously expands and deepens the research frontiers of information theory itself. Looking forward, we envision semantic information theory not only as a bridge between these two domains but also as a source of practical insights and rigorous methodologies that will inform the design of next-generation communication networks.
